# Drilling of Hybrid Titanium Composite Laminate (HTCL) with Electrical Discharge Machining

**DOI:** 10.3390/ma9090746

**Published:** 2016-09-01

**Authors:** M. Ramulu, Mathew Spaulding

**Affiliations:** Department of Mechanical Engineering, University of Washington, Seattle, WA 98195, USA; mspaulding@uw.edu

**Keywords:** EDM, HTCL, machinability, TiGr, thermoplastic composites

## Abstract

An experimental investigation was conducted to determine the application of die sinker electrical discharge machining (EDM) as it applies to a hybrid titanium thermoplastic composite laminate material. Holes were drilled using a die sinker EDM. The effects of peak current, pulse time, and percent on-time on machinability of hybrid titanium composite material were evaluated in terms of material removal rate (MRR), tool wear rate, and cut quality. Experimental models relating each process response to the input parameters were developed and optimum operating conditions with a short cutting time, achieving the highest workpiece MRR, with very little tool wear were determined to occur at a peak current value of 8.60 A, a percent on-time of 36.12%, and a pulse time of 258 microseconds. After observing data acquired from experimentation, it was determined that while use of EDM is possible, for desirable quality it is not fast enough for industrial application.

## 1. Introduction

Hybrid Titanium Composite Laminates (HTCL) possess unique mechanical and thermal properties such as high specific strength and specific modulus, and superior strength at elevated temperature which makes them widely used for applications in high temperature components in the aerospace field [1]. However, the difference in machinability of the titanium alloy and graphite composite components present difficulties in machining titanium graphite (TiGr) laminates with conventional methods [2,3,4]. Some examples of machining difficulties include: extremely high tool wear rate caused by the abrasive nature of the reinforcement fibers, delamination, fiber pullout, and titanium burrs. Specifically, conventional drilling of HCTL has been seen to yield defects in the form of voids, poor surface quality on the interior surface of drilled holes, delamination of the face sheet material, and backside rupture of the Titanium lower face sheet [5,6,7].

Electrical Discharge Machining (EDM) has proven itself a capable method for machining a variety of materials [4,8] and was successfully applied in machining titanium [9,10,11,12,13], metal matrix composites [14,15,16,17] as well as ceramic composites [4,18,19,20,21]. The ability of EDM to remove material without application of mechanical force has the possibility of eliminating many of the difficulties seen in conventional machining of composite materials such as drilling. Research into the proper settings for EDM of polymer matrix composites (PMCs) has shown that there is a very specific range of settings at which the workpiece can be machined [4,22,23,24,25,26,27]. Ordinarily, for these materials, the matrix material is non-conductive; however, if the fiber material is conductive, the fibers can be used to generate sparks required for material removal by EDM. Positive polarity was found to produce minimum tool wear thereby increasing the quality of cuts in a polymer composite material. At higher currents (approximately 5 A and higher) defects such as undesirable thermal expansion of the fibers within the composite can result causing hexagonal shaped fibers, debonding between the fibers and the matrix material, as well as severe melting of the composite surface. At lower currents (<5 A), the quality of the features is commonly much higher [22,23,24], fibers will more likely remain in an undisturbed state, and the heat affected zone surrounding the feature is usually greatly decreased. Along with current settings, the shape of the current pulse can also affect the quality of the features created [23]. Varying the lengths of time dedicated for sparking and those dedicated for flushing the molten workpiece material can have significant effects on cutting time as well as surface finish. Having too long a delay between sparks can result in lengthened cutting times as well as the possibility of shorting between the workpiece and tool [26,27]. If the pulse delay is too short, the material will not have adequate time to be properly flushed, which can result in material being deposited on the tool, or recast on the workpiece. Either of which will decrease the machining efficiency, as well as decrease the quality of the cut. These defects can be avoided if the energy used for processing is kept low enough; however, this was noted to extend the cutting time [21,22,24,26,27]. In summary, when properly applied, it has been demonstrated that composite materials can be machined using EDM [28]. However, EDM applied to hybrid thermoplastic composite materials has not yet been researched. This paper presents the first results of using EDM to produce holes in TiGr.

The objective of this paper is to investigate the use of EDM on a particular type of HTCL, known as TiGr (Titanium Graphite). TiGr, as used in this study, is comprised of two outer plies of titanium sheets that sandwich a composite core. The composite core is a high-temperature thermoplastic matrix material, PIXA-M, that is reinforced with IM-6 graphite fibers. Able to withstand temperatures up to 177 °C, the core is intended to provide good fatigue resistance and improve the strength to weight ratio, while the outer layers of titanium protect the PIXA-M from environmental weather concerns and from impact. The workpiece material removal rate (MRR), tool material removal rate or tool wear rate (TWR), surface roughness, and quality hole geometry are the four important machinability criteria with economic implications. To date, no research has been conducted that characterizes EDM machinability of this material. Therefore, the purpose of this study is to evaluate EDM machinability by varying EDM process conditions, namely, the effect of peak current, pulse time, and percent on-time on work piece material removal rate, tool wear rate, and surface quality.

## 2. Experiment Setup and Methodology

**Figure 1:**
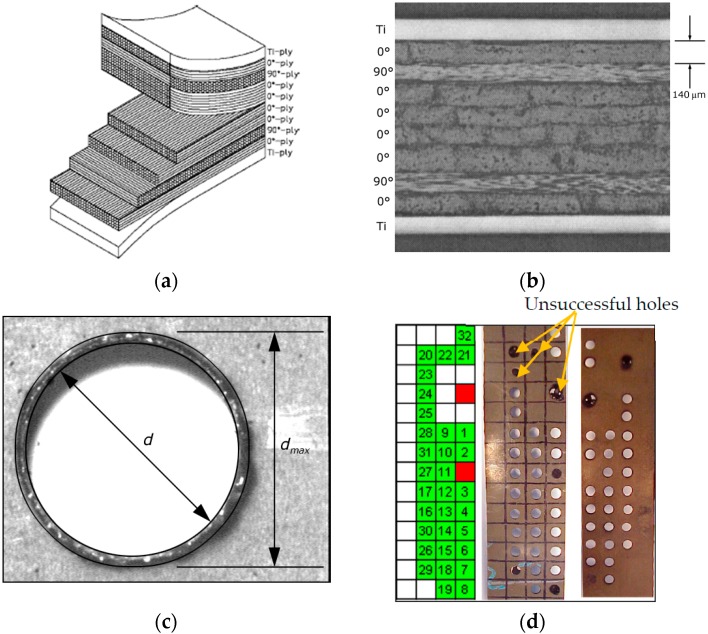
(**a**) Schematic of the TiGr laminate studied; (**b**) TiGr laminate specimen; (**c**) Definition of hole damage geometric parameters; (**d**) Die-sinker hole layout.

A TiGr workpiece with dimensions 304.8 mm × 304.8 mm × 1.4 mm was utilized in this investigation with details of the autoclave consolidation process used for fabrication described in references [1,2]. A 50.8 mm × 304.8 mm test specimen of this material was sectioned utilizing a diamond coated abrasive band saw blade. The TiGr workpiece is made up of two surface plies of Ti with eight plies of PIXA-M thermoplastic polyimide resin (Mitsui Chemicals, Tokyo, Japan) and IM7 intermediate modulus graphite fiber (Hexcel, Yokohama, Japan), with a ply thickness of approximately 140 micrometers. The continuous fibers in the PIXA-M had an approximate diameter of 6 micrometers. These plies were laid out with the following symmetric stacking sequence: {Ti,(0, 90, 0, 0, 0, 0, 90, 0),Ti} as shown both schematically (Figure 1a) and with a photomicrograph (Figure 1b) in Figure 1. The resistivity of the carbon fibers is 9.5–18 µΩ·m. Matrix material’s resistivity is less than 1 × 10^−6^ ohm^−^·m and titanium have a resistivity of 1.47 × 10^−6^ ohm^−^·m [28,29,30]. The exact conductivity or resistance depends on the modulus of the fiber. In general, high modulus fibers have lower resistance and low modulus fibers have higher resistance. Typical material properties for the TiGr material used in this investigation are given in Table 1.

The tool material used in the experiments was 6.35 mm diameter copper 110 H04 round stock. Copper electrode material was selected based on the reported machining efficiency and minimum tool wear in drilling titanium [9,10,11] and polymer composites [22,24,25,26,27]. The tool density was 8.91 gm/cm^3^ with a melting point of 1083 °C. Copper was chosen due to its low electrode wear rate, high quality surface finish, and material removal rate (MRR) when applied to EDM of graphite composite materials, as well as its low cost and its ease of availability. A Hansvedt Benchman EDM M-Pulse CNC machine (Hansvedt, Chicago, IL, USA) was utilized for the experiments. The dielectric fluid used in this machine was Rust-Lick EDM-250 (Hansvedt, Chicago, IL, USA), which has a dielectric strength of 47 kV and a viscosity of 0.01 P. The workpiece and tool were both submerged in the EDM tank, and two nozzles at 137.9 kPa placed approximately 12.7 mm from the spark gap flushed the cut.

Spark energy is the product of peak current, percent on-time and pulse duration. Since these process variables can be readily adjusted, machining conditions using positive polarity, were selected based on the reported literature for polymer composites [23,24,25,26,27,28]. Hence, these three principle EDM parameters were selected for this study also and the experiments were established upon full factorial design of these parameters. In order to determine optimal values for drilling the TiGr material, a 3-factorial design of experiments (DOE) was setup using Design Expert 6 software [32]. Factors and levels are given in Table 2. The makeup of this experimental design consisted of three variables, namely, peak current in amperes (A), percent on-time (duty factor = t_on_/(t_on_ + t_off_)), and pulse time in microseconds (µs); each with three levels. The machining parameters and drilling conditions were chosen based on literature [8,9,10,11,12,20,21,22,23,24,25], and compromised selection. Past reported studies showed that pulse geometry and magnitude were the most relevant parameters to achieve desirable features using EDM. The remaining variables capable of being adjusted were kept constant throughout the experimental runs through machine settings. Machine settings used in the experiments are given in Table 3. The responses considered are workpiece material removal rate, tool wear rate, surface quality, and damage. The experimental design employed is given in Table 4.

Material Removal Rate (MRR) is defined as the volume of material removed per unit cutting time. Tool wear rate (TWR) is defined as the change in volume of the tool material per unit cutting time. The volume change was calculated by determining the tool’s mass prior to and following the drilling process on a Mettler AE240 Scale (Mettler, Oakland, CA, USA). With known density of the tool material, the change in tool volume can be determined. Sinker EDM hole quality was evaluated in terms of taper, damage ratio, recast layer, and surface roughness. The damage ratio is defined as (d_max_/d) where d_max_ is the damaged diameter and d is the hole diameter as defined in Figure 1c. EDM hole production layout is shown in Figure 1d. Measurement was conducted utilizing black and white micrographs of each drilled hole. The discoloration and burr formation on the titanium surface are used to define the damage diameter with measurement process similar to the method used by Guu et al. [22].

Following the damage ratio measurement, the specimen was sectioned exposing the interior surfaces of the holes. An optical microscope was used to take macrographs of the interior surfaces of each of the holes at 50× magnification. The macrographs of the drilled samples were analyzed for any visible damage to the material. From these macrographs, the highest quality hole and lowest quality hole were selected and set aside for imaging using a scanning electron microscope (SEM). After being imaged with the SEM, average roughness measurements were taken of all of the samples using a Taylor-Hobson Surftronic 3 (Taylor Hobson, Leicester, UK) with a cutoff of 0.01 microinches and a traverse distance of 0.5 mm.

Based on a design of experiments approach, a statistical analysis of variance (ANOVA) is performed in order to determine the effects of each of the variables that are statistically significant on each of the responses analyzed. In order to develop a statistical model of machining behavior of the cuts, measured data was examined and fitted using a multiple linear regression model of the form: in which the following are the coefficients:

(1) $$y\left(x\right)=C_{0}+\underset{i}{\sum }C_{i}x_{i}+\underset{i}{\sum }C_{ii}x_{i}^{2}+\underset{j>i}{\sum }\sum \;C_{ii}x_{i}x_{j}$$

$C_{0}$ = Constant; $C_{i}$ = First order or linear effect; $C_{ii}$ = Second order or quadratic effect; $C_{ij}$ = Interaction effects.

The variables are the parameters and denoted as follows: $x_{1}$ = Pulse time (micro seconds); $x_{2}$ = Percent on time (% micro seconds); $x_{3}$ = Peak current (ampere).

The response equations for Workpiece Material Removal Rate, Tool Removal Rate or Tool Wear Ratio, Surface Roughness and Damage Ratio, follow in the next section. Depending on the model used to fit the data, individual effects and interaction effects can be determined as significant with respect to each response. Once these are determined, an equation is generated using the various effects to fit the specified model.

## 3. Results

**Figure 2:**
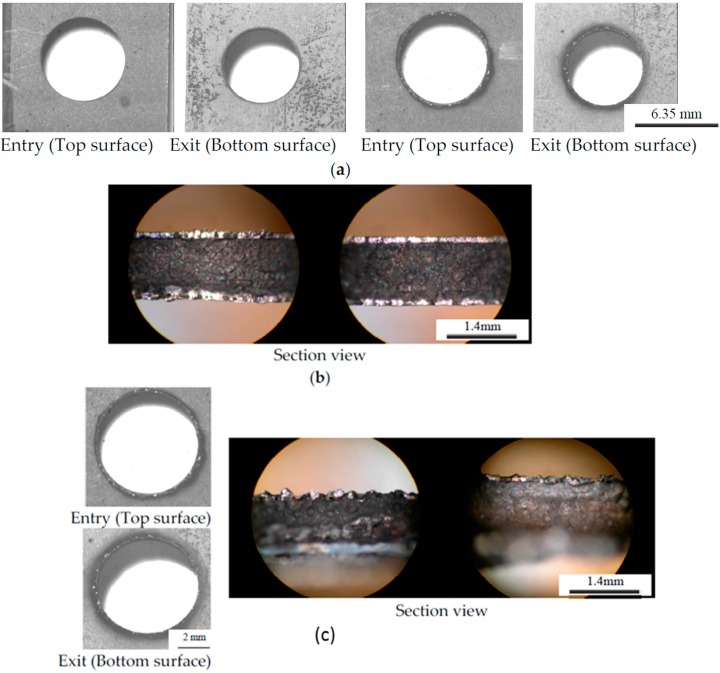
Typical Optical Micrographs of the EDM machined high quality, and poor and failed holes: (**a**) Photo macrographs of Electrode Entry (Top Surface) and Exit (Bottom Surface) of Hole-1 (**left**, 25 µs, 50%, 2 A) and Hole-2 (**right**, 400 µs, 75%, 11 A); (**b**) Cross-section of Poor quality Hole-31 (400 µs, 75%, 5 A); (**c**) Top Bottom and section view of failed Hole-20 (100 µs, 75%, 11 A).

Photo-macrograph of 32 holes drilled using a die sinker EDM is shown in Figure 1d. Typical high quality, poor, and failed holes are shown in the Figure 2 with the associated EDM machining conditions. Unlike conventional drilling the top and bottom titanium ply was free of burrs in quality hole (Hole-1) and damage and heat affected zone for poor ones (Hole-31, 20). Good quality holes were associated with peak current of 2 A as seen Figure 2a. It should also be noted that some of the holes were stopped prior to completion. Hole-8, 20 (Figure 2c), 22, and 23 were stopped due to buildup of re-deposited material resulting in a short between the workpiece and tool. Hole-29 was stopped due to the excessive time required. An example sample of very poorly machined Hole-31 (Figure 2b), and failed Hole-20 (Figure 2c) optical micrographs are shown in Figure 2. Note the thermal damage and heat effected zone (dark zone) in titanium layer both at the tool entry, exit positions. Severe damage can be seen in micrographs of sectioned hole walls Figure 2a,b. Cutting time was recorded for each holes cut, and not surprisingly, more rapid drilling was associated with those cuts which were performed at a higher peak current. Note that the 50% On-Time maintained the most rapid cuts through all current settings; as the peak current increased, the longer pulse time allowed for the higher volume of melted material to be more effectively removed.

### 3.1. Material Removal and Surface Topography

**Figure 3:**
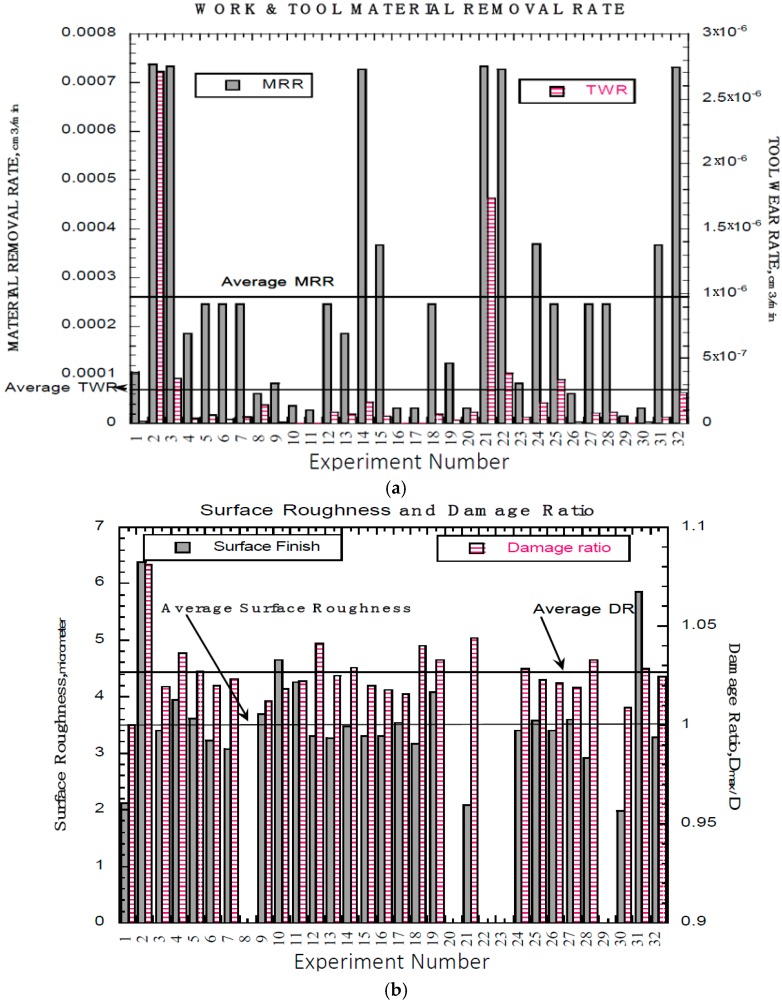
EDM Hole Machinability in terms of (**a**) Material Removal Rate (MRR) and Tool Wear Rate; (**b**) Surface Finish and Damage Ratio.

The machinability in terms of MRR, TWR, surface roughness and damage ratio data generated from the production of these holes was recorded and is presented in Figure 3. As can be seen Figure 3a, the holes-2, 3, 14, 21, 22, and 32 have produced high MRR and was found to be associated with higher peak current (11 A). At lower current (2 A), MRR was very low and the fibers were not disturbed regardless of its orientation to the cutting direction. Interestingly, MRR of holes-15, 24 and 31 was 3.67 × 10^−4^ cc/min. At maximum peak current with longer pulse time allowed for the higher volume of melted material to be more effectively removed. The maximum, average and minimum MRR was found to be 7.37 × 10^−4^, 2.6 × 10^−4^ and 2.73 × 10^−5^ cc/min respectively. In all experiments the TWR was very low with positive polarity regardless of the pulse on time and the % on-time, when the peak currents of 2 A and 5 A. Highest TWR was found to be 1.74 × 10^−6^ and 2.71 × 10^−6^ cc/min for the Holes-2 (400 µs, 75%, 11 A), and 21 (25 µs, 25%, 11 A) respectively as can be seen in Figure 3a. The lowest TWR of 1.90 × 10^−8^ cc/min, and it was for the Hole-1 (25 µs, 50%, 2 A).

The surface roughness and damage ratio vs. hole number or experiment number is shown in Figure 3b. The surface roughness ranged from 1.98–6.38 µm and damage ratio from 1.0–1.08, for all the experiments. The average surface roughness and damage ratio shown as solid straight line in Figure 3b are 3.5 mm and 1.03 respectively. As the peak current increased the surface roughness increased. All of these experimental observations are consistent with others [22,24,26].

**Figure 4:**
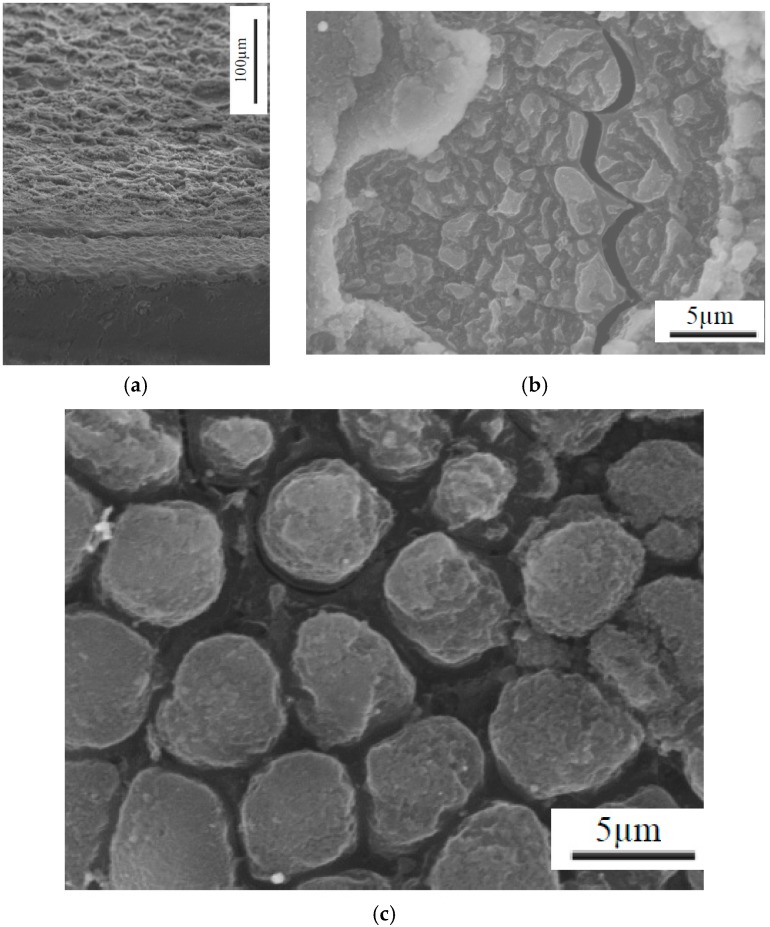
Typical SEM Micrographs of the EDM Machined TiGr Surface Features depicting: (**a**) Recast layer in titanium ply and composite core; (**b**) Matrix cracking; (**c**) Fiber expansion.

**Figure 5:**
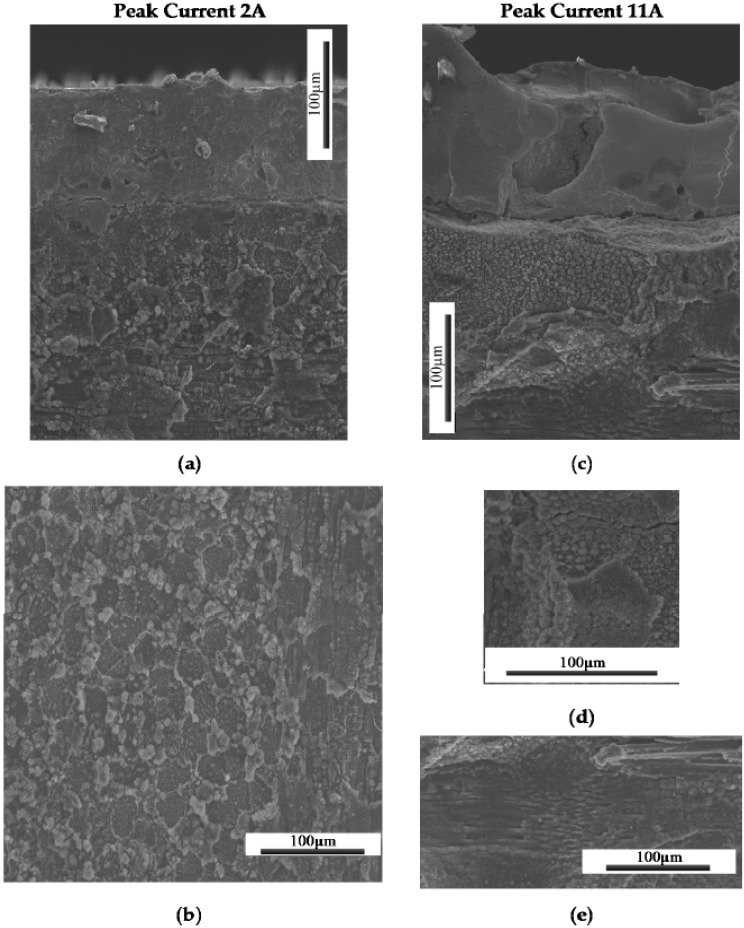
Typical SEM Micrographs of the EDM Machined TiGr Surface Features for 2 A peak current depicting (**a**) Interface of Ti and carbon fibers; (**b**) Interface between 0° and 90° plies; and for 11 A peak current depicting (**c**) Interface of Ti and carbon fibers; (**d**) 90° composite plies; (**e**) Interface between 0° and 90° plies.

Figure 4 shows the TiGr composite machining surface from the EDM process. Matrix material was melt faster than fiber and solidified. Note that the sectioned surface shown in Figure 4a, clearly shows craters, the recast layer in titanium ply and polymer core. From the examination of the surface characteristics, it was observed that EDM process produces much damage on the surface such as globules, varying size craters, cracks and debonding the plies. Thus the surface is uneven and can be seen with random size craters. Severe melting of the composite surface in particular matrix melting and debris re-solidifying along with cracks can be seen in Figure 4a. Recast layer in titanium ply was ranged from 1–10 µm revealing lowest lowest peak current of 2 A and higher at 11 A. It was also found that at higher energy pulse (peak current × pulse on time), thermal expansion of 90 degree fiber shown in Figure 4c were laterally expanded and was consistent with others [24]. As the peak current and percent of on time increased, the deeper craters were more evident and rougher surfaces were more pronounced. The topography and typical characteristic features examined with SEM and shown in Figure 4 and Figure 5. Figure 5 clearly show the effect of current on the surface topography and quality of the machined surface. In addition to the peak current, short pulse on time produced a finer finish and peak current long pulse time produced a rougher surface.

Design Expert [32] was used to generate ANOVA and analyzed the data for the significance of input parameters for each process variable with Equations (2)–(5) obtained for predicting Workpiece MRR, TWR, Surface Roughness, and Damage Ratio, respectively. Response plots were plotted and presented in the following sections for selected peak currents of 2, 5 and 11 A. In the analysis of the data from this research, the models fitting the data points for these 5 unfinished cuts were not included.

### 3.2. Response Surface Modeling

The results of ANOVA for the response functions workpiece MRR and TWR are given in the appendix—Table A1 and Table A2 respectively. ANOVA identified that peak current had a dominant effect, both in linear, and quadratic effect, as well as percent on-time’s quadratic effect as being the most significant. This agrees to the data shown in Figure 3. A regression fit through the data points using a quadratic surface with a log10 transform yielded Equations (2) and (3) for Workpiece MRR and TWR with an R^2^ value of 0.85 and 0.91 respectively.

Workpiece Material Removal Rate (*W_MRR_*)

(2) $$\begin{array}{ll} \log_{10}(W_{MRR})= & -6.70888-(2.74860\times 10^{-3})x_{1}+0.026479x_{2}+0.39114x_{3}\\ & +(2.42195\times 10^{-6})x_{1}^{2}-(2.59911\times 10^{-4})x_{2}^{2}-0.021078x_{3}^{2}\\ & +(1.65183\times 10^{-5})x_{1}x_{2}+(5.10268\times 10^{-5})x_{1}x_{3}+(1.01344\times 10^{-4})x_{2}x_{3} \end{array}$$

Tool Wear Rate (*T_MRR_*)

(3) $$\begin{array}{ll} \log_{10}(T_{MRR})= & -8.22530-(9.44304\times 10^{-3})x_{1}+0.020838x_{2}+0.349449x_{3}\\ & +(1.89694\times 10^{-5})x_{1}^{2}-(2.47282\times 10^{-4})x_{2}^{2}-0.029215x_{3}^{2}\\ & -(5.80415\times 10^{-6})x_{1}x_{2}-(9.94958\times 10^{-5})x_{1}x_{3}+(3.07325\times 10^{-3})x_{2}x_{3} \end{array}$$

**Figure 6:**
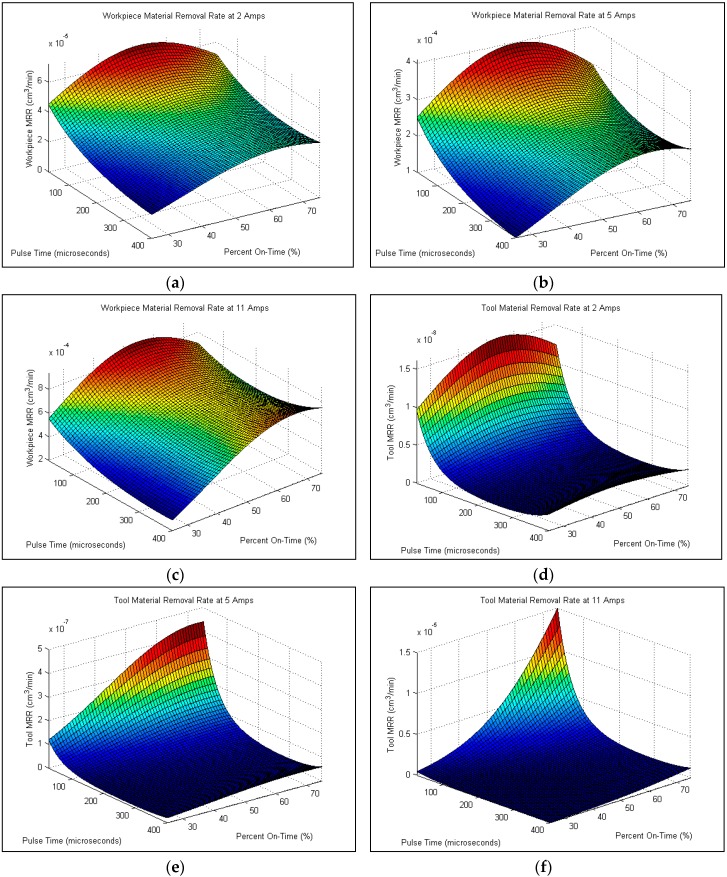
Effect of process parameters on workpiece MRR for (**a**) 2 A; (**b**) 5 A; (**c**) 11 A peak current and on TWR for (**d**) 2 A; (**e**) 5 A; (**f**) 11 A peak current.

Figure 6 shows the trends of MRR both in workpiece (Figure 6a–c) and tool material removal rate (Figure 6d–f). In general, workpiece MRR followed the trend set by the cutting time due to its dependence on those values. Since the MRR is calculated from cutting times, the highest MRR values were found at high currents, low pulse, and a 50% On-Time and is similar to the values found for a low cutting time. From Figure 6d–f, it can be seen that as peak current increased, the more uniform the tool wear rate, though the amount of wear also increased. The only tools which showed significant amounts of wear were found on the four cuts which did not finish due to shorting between the tool and workpiece. The omission of failed hole data in the generation of Equation (3) is the most likely reason for the spike found in Figure 6f.

Hole quality is presented in terms of machined hole surface and the surface damage. The results of ANOVA for the response functions surface roughness is shown in Table A3 and similar observation was made on damage ratio ANOVA and not listed in the Table A1 and Table A2. From ANOVA of average surface roughness, R_a_, and damage ratio, d_max_/d, it was found that Pulse Time and Percent On-Time were the significant factors on R_a_, with an (Equation (4)) and percent on-time and peak current damage ratio (Equation (5)). The surface roughness and damage ratio model equations were determined with an an R^2^ value of 0.98 and 0.70 respectively.

Surface Roughness (*R_a_*)

(4) $$R_{a}=1.837088+0.003894x_{1}+0.019521x_{2}+0.019321x_{3}$$

Damage Ratio (*D_f_*)

(5) $$\begin{array}{ll} D_{f}= & 1.030106-(1.2\times 10^{-5})x_{1}-0.00132x_{2}+0.004413x_{3}\\ & +(9.63\times 10^{-8})x_{1}^{2}+(1.16\times 10^{-5})x_{2}^{2}-0.0004x_{3}^{2}\\ & -(9.8\times 10^{-8})x_{1}x_{2}-(2.5\times 10^{-6})x_{1}x_{3}+(8.76\times 10^{-5})x_{2}x_{3} \end{array}$$

where $x_{1}$ relates to the pulse time in microseconds, $x_{2}$ is the Percent On-Time, and $x_{3}$ is the Peak Current.

**Figure 7:**
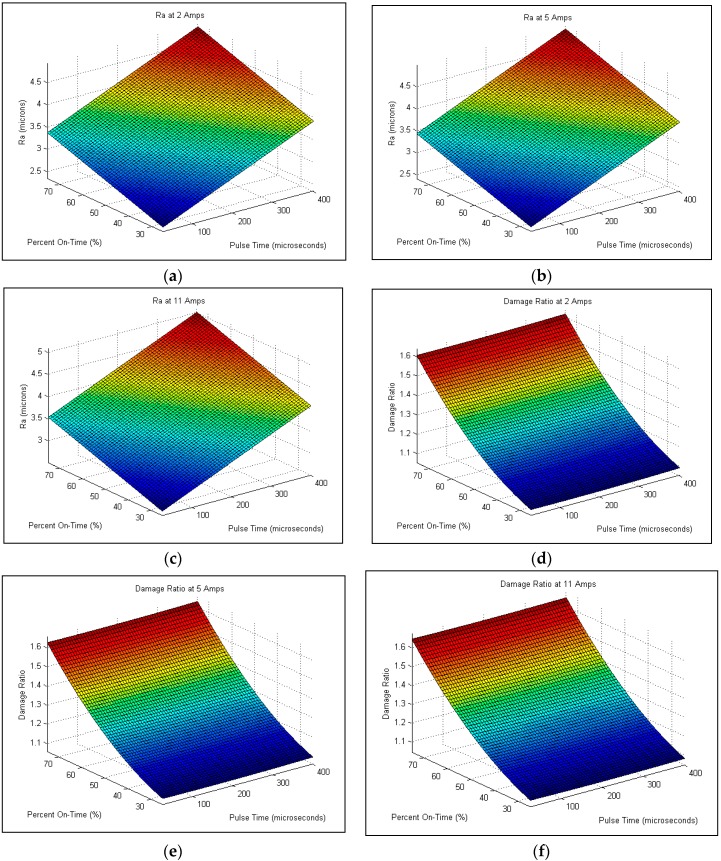
Effect of process parameters on surface roughness (R_a_) for (**a**) 2 A; (**b**) 5 A; (**c**) 11 A peak current and on damage ratio for (**d**) 2 A; (**e**) 5 A; (**f**) 11 A peak current.

Figure 7 depicts the process conditions on hole quality. It can be seen from Figure 7a–c that the variation of surface roughness between each of the individual currents was negligible; however; as Percent On-Time increased and Pulse Time increased, average surface roughness increased. While surface roughness increased as current increased, the change was very small. From Figure 7d–f, it is possible to see that pulse time does not contribute in any significant manner to the damage ratio of the drilled holes. However, as percent on-time increased, the damage ratio increased possibly due to increased molten material. The damage ratio also increases slightly with increasing current. This isn’t surprising as in many manufacturing processes there is a trade-off between damage and cutting time, where in this case the shortest cutting times occurred at high currents. One difficulty with this form of damage assessment is that it cannot account for thermal damage subsurface of the cut in the composite layers of the material.

## 4. Discussion

### 4.1. Effect of EDM Process Parameters

Effects of EDM process conditions on hole production in terms of MRR, and surface quality are presented in Figure 2, Figure 3, Figure 4, Figure 5, Figure 6 and Figure 7. It is possible to analyze the effects of each of the individual variables and from that analysis estimate optimized conditions for processing of TiGr composite. Through this analysis, it is possible to determine the process parameters which will result in the most rapid cut, the least amount of tool wear, or the highest amount of workpiece material removal.

#### 4.1.1. Effect of Peak Current

From Figure 2, Figure 3, Figure 4, Figure 5, Figure 6 and Figure 7, it can be seen that peak current has an effect on workpiece MRR, TWR, and damage ratio. In some cases, the most rapid 5 A cuts were able to approach cutting times relative to the slowest cuts of the 11 A holes, but overall the 11 A cuts were the fastest and can be seen in Figure 3. Of the holes drilled, the holes drilled at 11 A also had the highest rate of failure. Of the nine holes drilled at 11 A, three did not finish (Hole-8, 20 and 22) as seen in Figure 2. A combination of high current and inadequate time to clear melted material prevented proper flushing and resulted in failed cuts. Unfortunately, the trade-off with the more rapid cut is that the quality of the hole is significantly decreased. Shorter cutting times are directly associated with higher workpiece MRR which was also due to the increase in peak current. Between each of the peak current settings (2 A, 5 A, 11 A), tool MRR increased nearly by an order of magnitude, though the trend in general remained similar between the three settings as shown in Figure 6 and is consistent with others [23,24,25,26].

Tool MRR only slightly increased as the peak current increased. The only dramatic change in tool MRR occurred in the holes drilled with short pulse time and high percent on-time values. At these conditions, tool MRR rose rapidly compared to the slow increase found at the higher pulse times and lower percent on-times. Considering the drilled surface, peak current has an insignificant effect on surface roughness, and only a small effect on the damage ratio as shown in Figure 5. The damage ratio slightly increased as the peak current increased.

#### 4.1.2. Effect of Pulse Time

The effect of changing the pulse time variable of a cut is the first of two which determine the duration of time during which current is applied between the workpiece and the tool as well as the length of time for flushing melted material. The second variable is the percent on-time which will be discussed in the following section. Analysis of experimental results show that in the 2 A and 5 A cases, cutting time decreased as the pulse time decreased. This results from the more stable arc at shorter pulse times. It was not so in the 11 A case which resulted in a saddle shaped surface with minimum cutting times at the high and low values explored for pulse time with maximum values at approximately 300 μs. Workpiece MRR followed a similar trend in the 2 A and 5 A cuts as was found in cutting time, in general increasing with a decrease in pulse time. The 11 A specimen again showed a saddle shape with the smallest workpiece MRR occurring around a pulse time of 300 μs. From Figure 4, Tool MRR also increased with a decrease in pulse time. This increase was very gradual from approximately 400 μs down to around 100 μs, where the tool MRR began to increase at an elevated rate. The effect of pulse time on the 11 A cut was very slight except in the combination of a short pulse time and a high percent on-time where tool MRR increased significantly. For the drilled surface, as the pulse time increased, the surface roughness increased as seen in Figure 5. This is due to the bigger molten area per spark resulting in a larger crater being created. However, pulse time has insignificant effect on damage ratio.

#### 4.1.3. Effect of Percent On-Time

Percent on-time is the second half of what determines how long current is applied. Short percent on-times cause smaller amounts material to be heated and allow more time for material to be flushed out of the workpiece tool gap, whereas longer percent on-times enable more material to become molten which can in turn increase MRR. The extended on-times cause a reduction in the flush time between sparks, which can result in inadequate removal of material from the gap, causing shorting or excessive material buildup on the tool. It was found that the effect of percent on-time on cutting time for the 2 A and 5 A cases was inversely related as can be seen in Figure 2. The 11 A case however was determined to have a minimum cut time at approximately 50% on-time, with longer cutting times as percent on-times increased. Most likely this was due to excessive buildup of matrix material on the tool surface. Cutting times also increased in the 11 A cases when the percent on-time was decreased from 50%. This trend is most likely due to the smaller volume of material which is melted per spark. In general, workpiece MRR increased with an increase in percent on-time; however, at higher percent on-times the amount at which MRR increased slowed, and at lower pulse times actually decreased slightly. The 11 A workpiece MRR actually decreased at all pulse times higher than a percent on-time of approximately 50%. Tool MRR increased with rising percent on-times, with maximum values slightly higher than 50% for the 2 A cuts and 70% for the 5 A cuts. The 11 A cuts had a relatively constant tool MRR except for at the highest limit for the percent on-time and lowest pulse time. At this point, tool MRR reached its maximum, nearly an order of magnitude above the values for the rest of the 11 A cuts. Percent on-time also has a significant effect on surface roughness and damage ratio as can be seen from Figure 5. Increasing percent on-time results in a rougher surface and larger damage ratio. This is due to inadequate flushing and excessive heating.

Optimization of cutting parameters was performed using Design Expert. For application in industry, the most favorable result for a drilled hole would take place in a short cutting time, achieving the highest workpiece MRR, with very little tool wear. With these values supplied to the software, the optimal settings for a hole drilled were determined to occur at a peak current value of 8.60 A, a percent on-time of 36.12%, and a pulse time of 258.01 μs. According to these values, the resulting cut would be finished in just slightly over an hour at 62 min. Workpiece MRR would be maximized at 4.764 × 10^−4^ cc/min with tool MRR minimized at 7.129 × 10^−8^ cc/min. These values reflect the most desirable material processing conditions with one difficulty.

**Figure 8:**
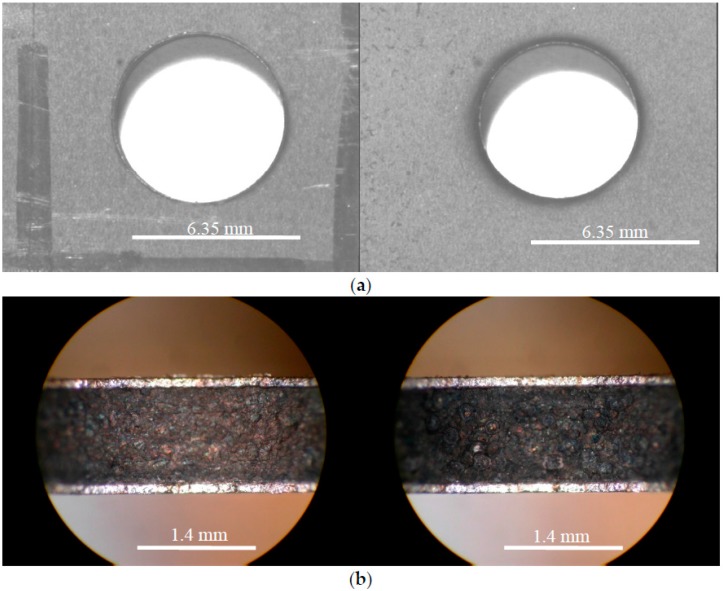
Macrographs of Hole-19 produced with near optimal EDM conditions, depicting (**a**) Top and bottom views; (**b**) Sectioned view at 50× resolution.

In general, the tradeoff for a fast cutting time and high workpiece MRR is a decrease in surface quality. Hole-19, the closest hole cut to the optimized process parameters can be seen in Figure 8. These top and bottom hole surface macrographs (Figure 8a) and sectioned hole surface optical micrographs (Figure 8b) show that while the hole quality is fair, the resulting surface from the cut contains slight damage and discoloration to the Ti plies as well as some pitting in the composite internal plies. This damage could result in a decrease in the lifetime of products manufactured using the optimized parameters.

### 4.2. Surface Quality

**Figure 9:**
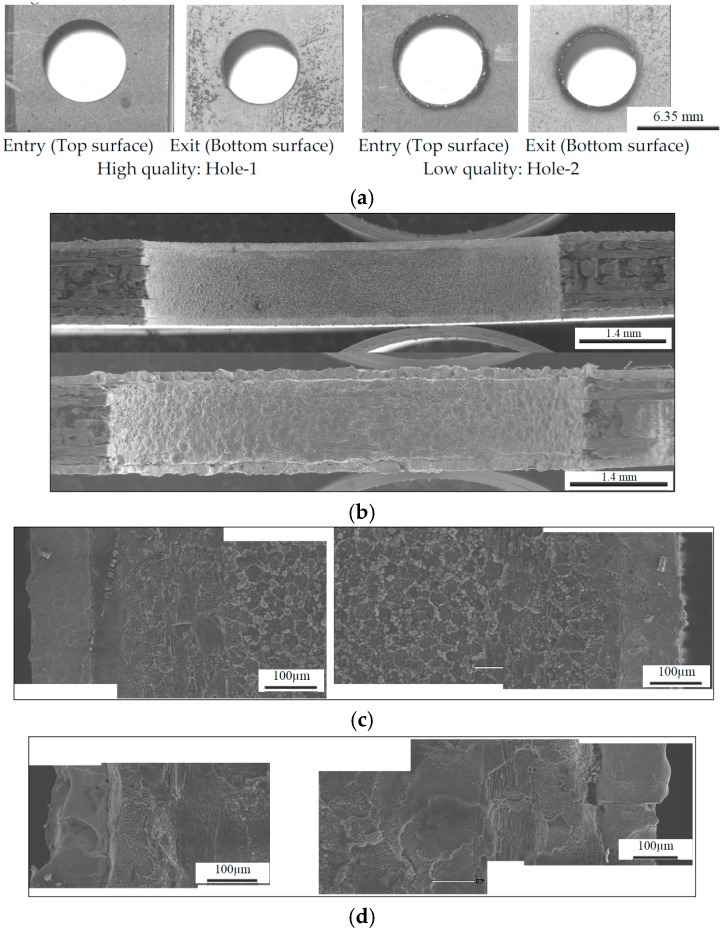
Surface Topography SEM Micrographs of the sectioned high and low quality holes: (**a**) Electrode Entry (Top Surface) and Exit (Bottom Surface) of Hole-1 (**left**) and Hole-2 (**right**); (**b**) Sectioned SEM Micrographs of Hole-1 (**top**) and Hole-2 (**bottom**); (**c**) Surface Topography of high quality Hole-1; (**d**) Surface Topography of low quality Hole-2.

Using an optical microscope, the general trend witnessed was that the lower peak current cuts resulted in a much cleaner surface. The craters were shallow and blended resulting in an overall relatively uniform smooth surface. Many of the cuts at higher currents, including many of the 5 A cuts and all of the 11 A cuts, showed heat damage and highly cratered surfaces including sections where matrix material was extruded from around the ends of the graphite fibers thereby leaving bare fibers exposed. Figure 9a shows an SEM micrograph of the selected highest quality and lowest quality holes cut using the die sinker EDM. It is obvious that the lower quality hole is significantly rougher than the high quality cut. This was supported by the average roughness (R_a_) measurements taken of the drilled specimens. More in depth analysis of Hole-1 was performed with an SEM. As can be seen in Figure 9b, the surface contains a great deal of recast matrix material; however, both Ti layers remain intact and very little inter-ply delamination is evident. Furthermore, the individual fibers within the cut remain undisturbed, though in some locations small cracks in the matrix material were found. SEM micrographs were also taken of the poorest quality hole drilled for comparison. Figure 9b shows the SEM micrograph of Hole-2. Unlike Hole-1, there is extensive damage to not only the Ti plies, but also to the interior composite plies as well. There is evidence of recasted matrix material as observed by Lau et al. [23], and the craters seen in this specimen are large enough to result in a highly irregular surface.

**Figure 10:**
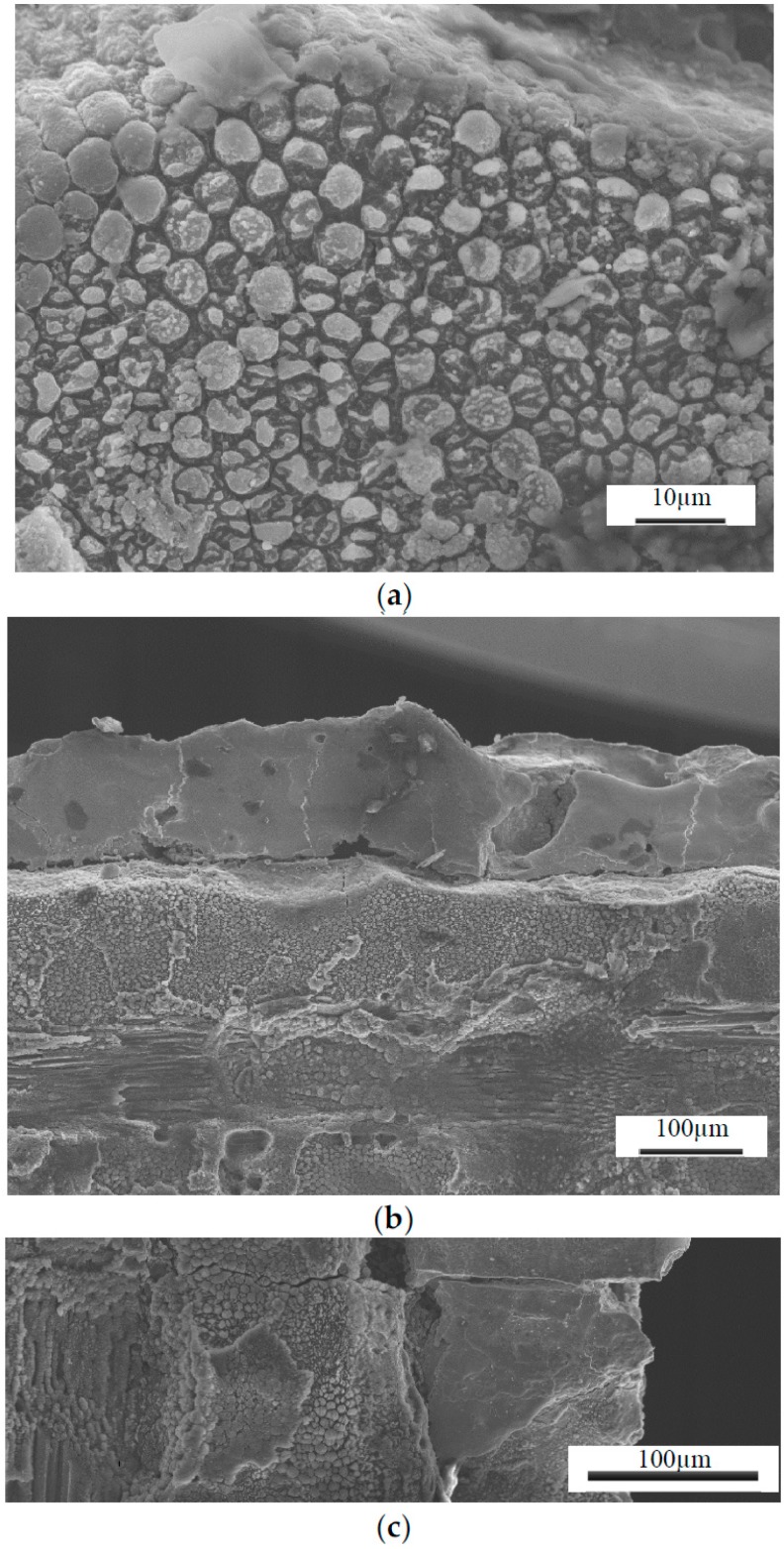
Typical EDM machined Titanium-Graphite composite material surface features depicting: (**a**) Matrix Extrusion Resulting in Exposed Fiber Ends; (**b**) Inter-ply debonding between Ti and composite plies; (**c**) Cracking through multiple layers of both titanium as well as graphite composite.

Evidence can also be seen of exposed, deformed, and fractured fibers as shown in Figure 10. On a larger scale, damage in the form of inter-ply delamination between the Ti and composite plies can be seen in Figure 10a. Overall, the quality improvement over conventional methods is not significant enough to warrant application of the EDM process. The additional plies of Ti were not enough to prevent the damage seen by previous researchers to the composite material. The damaged hexagonal fibers seen by others [22,23,24,25,26] were still found, as well as the melted matrix material and fiber matrix debonding. The main difficulty found in application of die sinker EDM to TiGr stems from the difference in machinability between the Ti and composite materials. Previous research has shown that polymer matrix composite (PMC) materials require small currents and short pulse on-times in order to produce acceptable cuts; however, Ti requires higher currents than the composite material to be machined in a timely manner. This leads (based on face sheet titanium work conducted) to one possibility for future research with fiber metal laminates using more metal layers and the experiments are in progress and will be reported in future.

## 5. Conclusions

The research performed analyzed the feasibility of applying Die Sinker EDM technologies to the processing of TiGr hybrid laminates. Holes were drilled through a specimen according to an experimental test matrix designed using a 3 factorial DOE in order to determine the effect of peak current, pulse time, and percent on-time on the results of the finished surface. The drilled specimens were sectioned and observed through an optical microscope in order to determine damage characteristics and the general quality of the holes. The highest and lowest quality holes were then analyzed utilizing an SEM in order to further study the condition of the cut surfaces. From this research, the following conclusions can be reached:

- Utilizing proper settings it is possible to machine TiGr using EDM methods.
- Higher currents will result in higher material removal rate; however they also adversely affect the surface of the cut.
- Damage due to improper process parameters can result in excessive material cracking, matrix burning and extrusion, as well as inter-ply delamination between composite and composite-metal plies.
- Using currents which are too high can result in failed cuts due to the inability of molten material to be properly flushed from the tool-workpiece gap.
- The highest quality holes occurred at the lowest current settings; however, the amount of time required in order to finish cuts at this setting exceed reasonable manufacturing production times.

## Figures and Tables

**Table 1:** Mechanical properties of the constituent materials of titanium graphite (TiGr) [31].

Properties	Material		
	Ti-15-3	LARC-IAX	IM7 Carbon Fiber
Longitudinal modulus (GPa)	100.61	3.33	272.2
Transverse modulus (GPa)	–	–	13.58
Shear modulus (GPa)	33.75	1.25	200
Poisson’s ratio	0.36	0.33	0.25
Yield strength (MPa)	1307	67.92	–
Ultimate strength (MPa)	1459	105	36,612
Coefficient of thermal expansion (10^−6^/k)	8.21	10	–
Percent elongation (%)	4.1	6	1.8

**Table 2:** Parametric factors and levels.

Factors	Level 1	Level 2	Level 3
Pulse On-time (µs)	25	100	400
% On-Time	25	50	75
Peak Current (A)	2	5	11

**Table 3:** EDM machine settings.

Parameter	Value
Gap Spacing	4
Servo Speed	1
Cutoff	3
Fault Retract	5
Cycle-EDM	0
Cycle-Retract	0
Capacitor (µF)	0
Pulse Type	M-Pulse
Tool Polarity	Positive
Tool Material	Cu
Workpiece Material	TiGr
Flush	on

**Table 4:** Experimental Design.

Test Number	Experiment/Hole Number	Pulse (µs)	% On-Time	Peak Current (A)
1	30	25	25	2
2	7	25	25	5
3	21	25	25	11
4	16	100	25	2
5	6	100	25	5
6	15	100	25	11
7	29	400	25	2
8	19	400	25	5
9	5	400	25	11
10	1	25	50	2
11	25	25	50	5
12	8	25	50	11
13	9	100	50	2
14	12	100	50	5
15	3	100	50	11
16	10	400	50	2
17	4	400	50	5
18	14	400	50	11
19	26	25	75	2
20	23	25	75	5
21	22	25	75	11
22	17	100	75	2
23	28	100	75	5
24	20	100	75	11
25	11	400	75	2
26	31	400	75	5
27	2	400	75	11
28	32	100	50	5
29	27	100	50	5
30	24	100	50	5
31	13	100	50	5
32	18	100	50	5

## Appendix A

**Table A1:** ANOVA for Workpiece Material Removal Rate.

Source	Degree of Freedom	Sum of Squares	Mean Squares	*F*-Values	Critical *F*-Values
Pulse	1	0.1023	0.1023	3.5319	0.0775
Percent On-Time	1	0.1284	0.1284	4.4330	0.0504
Peak Current	1	3.7843	3.7843	130.6994	<0.0001
Pulse^2^	1	0.0133	0.0133	0.4582	0.5076
Percent On-Time^2^	1	0.1377	0.1377	4.7568	0.0435
Peak Current^2^	1	0.7656	0.7656	26.4416	<0.0001
Pulse * Percent On-Time	1	0.0341	0.0341	1.1784	0.2928
Pulse * Peak Current	1	0.0105	0.0105	0.3629	0.5549
Percent On-Time * Peak Current	1	0.0006	0.0006	0.0210	0.8864
Residual	17	0.4922	0.0290	–	–

**Table A2:** ANOVA for Tool Material Removal Rate.

Source	Degree of Freedom	Sum of Squares	Mean Squares	*F*-Values	Critical *F*-Values
Pulse	1	1.7299	1.7299	21.3157	0.0002
Percent On-Time	1	1.3069	1.3069	16.1042	0.0009
Peak Current	1	13.0632	13.0632	160.9655	<0.0001
Pulse^2^	1	0.7988	0.7988	9.8424	0.0060
Percent On-Time^2^	1	0.1242	0.1242	1.5302	0.2329
Peak Current^2^	1	1.4702	1.4702	18.1154	0.0005
Pulse * Percent On-Time	1	0.0043	0.0043	0.0525	0.8215
Pulse * Peak Current	1	0.0405	0.0405	0.4988	0.4896
Percent On-Time * Peak Current	1	0.5033	0.5033	6.2020	0.0234
Residual	17	1.3796	0.0812	–	–

**Table A3:** ANOVA for Tool Material Removal Rate.

Source	Degree of Freedom	Sum of Squares	Mean Squares	*F*-Values	Critical *F*-Values
Pulse	1	8.1908	8.1908	22.6333	<0.0001
Percent On-Time	1	2.9060	2.9060	8.0300	0.0094
Peak Current	1	0.0921	0.0921	0.2544	0.6188
Residual	23	8.3235	0.3619	–	–
